# Correlation between Biophysical Properties of Niosomes Elaborated with Chloroquine and Different Tensioactives and Their Transfection Efficiency

**DOI:** 10.3390/pharmaceutics13111787

**Published:** 2021-10-26

**Authors:** Myriam Sainz-Ramos, Ilia Villate-Beitia, Idoia Gallego, Nuseibah AL Qtaish, Margarita Menéndez, Laura Lagartera, Santiago Grijalvo, Ramón Eritja, Gustavo Puras, José Luis Pedraz

**Affiliations:** 1 Laboratory of Pharmacy and Pharmaceutical Technology, NanoBioCel Research Group, Faculty of Pharmacy, University of the Basque Country (UPV/EHU), Paseo de la Universidad 7, 01006 Vitoria-Gasteiz, Spain; miriam.sainz@ehu.eus (M.S.-R.); aneilia.villate@ehu.eus (I.V.-B.); idoia.gallego@ehu.eus (I.G.); nusaiba.qtaish@gmail.com (N.A.Q.); 2 Biomedical Research Networking Centre in Bioengineering, Biomaterials and Nanomedicine (CIBER-BBN), Institute of Health Carlos III, Av. Monforte de Lemos 3–5, 28029 Madrid, Spain; sgrgma@cid.csic.es (S.G.); recgma@cid.csic.es (R.E.); 3 Bioaraba, NanoBioCel Research Group, Calle Jose Atxotegi, s/n, 01009 Vitoria-Gasteiz, Spain; 4 Rocasolano Physical Chemistry Institute, Superior Council of Scientific Investigations (IQFR-CSIC), Calle Serrano 119, 28006 Madrid, Spain; mmenendez@iqfr.csic.es; 5 Biomedical Research Networking Centre in Respiratory Diseases (CIBERES), Av. Monforte de Lemos 3–5, 28029 Madrid, Spain; 6 Institute of Medicinal Chemistry (IQM-CSIC), Calle Juan de la Cierva 3, 28006 Madrid, Spain; l.lagartera@iqm.csic.es; 7 Institute of Advanced Chemistry of Catalonia (IQAC-CSIC), Calle Jordi Girona 18-26, 08034 Barcelona, Spain

**Keywords:** chloroquine, niosomes, gene delivery, biophysical properties, surfactants

## Abstract

Lipid nanocarriers, such as niosomes, are considered attractive candidates for non-viral gene delivery due to their suitable biocompatibility and high versatility. In this work, we studied the influence of incorporating chloroquine in niosomes biophysical performance, as well as the effect of non-ionic surfactant composition and protocol of incorporation in their biophysical performance. An exhaustive comparative evaluation of three niosome formulations differing in these parameters was performed, which included the analysis of their thermal stability, rheological behavior, mean particle size, dispersity, zeta potential, morphology, membrane packing capacity, affinity to bind DNA, ability to release and protect the genetic material, buffering capacity and ability to escape from artificially synthesized lysosomes. Finally, in vitro biological studies were, also, performed in order to determine the compatibility of the formulations with biological systems, their transfection efficiency and transgene expression. Results revealed that the incorporation of chloroquine in niosome formulations improved their biophysical properties and the transfection efficiency, while the substitution of one of the non-ionic surfactants and the phase of addition resulted in less biophysical variations. Of note, the present work provides several biophysical parameters and characterization strategies that could be used as gold standard for gene therapy nanosystems evaluation.

## 1. Introduction

Gene therapy is based on the modification or control of gene expression in order to treat a specific disease [1]. There are two main gene carrier systems, viral and non-viral vectors, which provide genetic material protection and enhance cell internalization [2,3]. Non-viral vectors are usually based on different biocompatible nanoparticles, which represent a safer strategy than viruses and their elaboration process is easier and cheaper. Although in many occasions non-viral vectors are still unable to reach the high transfection levels of viral counterparts, continuous advances in the field bring us closer to this goal [4,5,6,7]. In fact, currently some lipid nanoparticles are already commercialized for drug and RNA delivery, such as Onivyde^®^ [8] and Onpattro™ [9], among others. In addition, during the ongoing pandemic caused by the severe acute respiratory syndrome coronavirus 2 (SARS-CoV-2) infection, two lipid nanoparticle-formulated mRNA vaccines have also been developed [10]. However, to date, non-viral vectors have not reached clinical practice for DNA delivery. To this end, it is essential the carefully design and selection of biomaterials to develop efficient nanocarriers with high biophysical performance.

Non-viral vectors can be classified on a wide variety of nanosized materials, including cationic lipids, polymers and carbon-based nanostructures [3,11]. Regarding cationic lipids, niosomes, which can be defined as vesicular structures made of non-ionic surfactants [12], have gained attention over liposomes due to their lower costs, longer stability and lower toxicity [13,14,15,16]. Basically, cationic niosome formulations for gene delivery applications [17] contain a cationic lipid, which forms complexes with the negatively charged DNA and promotes the fusion with cell membranes [18], non-ionic surfactants to form stable emulsions and prevent particle aggregations [7] and “helper” components to enhance the biophysical properties [19]. Cationic lipids have three main functional domains: a hydrophilic head-group which enhances electrostatic interactions with the DNA and can be formed by quaternary ammoniums, amines, amino acids, lysine, guanidiniums and heterocycles [20], a hydrophobic domain usually composed of two saturated/unsaturated aliphatic chains [21] and a linker bond which influences in the stability, biodegradability, transfection efficiency and cytotoxicity of the cationic lipid [22]. This linker bond usually contains an ester, ester amide, carbamate, disulphide, urea or phosphate, among others [22]. Some cationic lipids are commercially available, such as 1,2-di-O-octadecenyl-3-trimethylammonium propane (DOTMA) [7] or 1,2-dioleoyl-3-trimethylammonium-propane (DOTAP) [23], while others can be tailor-synthesized for specific applications. Regarding non-ionic surfactants, the most commonly used are polysorbates (Tween^®^) [7], sorbitan fatty acid esters (Span^®^) [24] and polyoxyethylene alkyl ethers (Brij^®^) [25]. The chemical structure of these surfactants influences on the final product as well as on their hydrophilic-lipophilic balance (HLB), which determines the oil or water solubility. For instance, the surfactants with long alkyl chains usually produce larger vesicles with more rigidity and less deformable membranes [7,26]. The role of the “helper” component is to increase the stability and the fluidity of the lipid bilayer, and it works as an adjuvant of the transfection process since it enhances cellular uptake and intracellular trafficking [19,27,28]. Until now, “helper” components used in non-viral vectors are neutral lipids such as phosphatidylethanolamine (DOPE) [23], cholesterol, squalane, squalene [19] and lycopene [29], but, recently, other compounds have also been explored. In this sense, chloroquine has emerged as an interesting material for gene delivery due to its ability to promote endosomal escape, which constitutes a key limiting step in every transfection process [30]. Although some peptides—such as cell-penetrating peptides (CPPs)—and viruses have also been used to that end, chloroquine presents higher stability than CPPs and its use results less complexity compared to viruses as it is not a biological agent. In addition, the lower cost and easier scale-up production of chloroquine also contribute to increase its attractiveness as a promising material for gene delivery purposes. In this work, we hypothesize that chloroquine not only promotes endosomal escape, but it could also be involved in the modification of different biophysical parameters of the nanoparticle formulations, which might have a direct impact on the transfection process. 

Along with the components and their chemical structure, the final biophysical properties of the system also depend on the elaboration method and the molar ratios between the nanocarrier components and the genetic cargo, which ultimately lead to different biological behaviors of the nanocomplexes [18,31,32,33]. To elaborate niosomes, many procedures have been described [15,34,35] being the oil in water (o/w) emulsion technique one of the most widely used. In this method, two phases are involved, the aqueous and the organic one. The niosome components are dissolved in the corresponding phase depending on their solubility and, after that, both phases are mixed by sonication to elaborate the emulsion. Thereupon, the organic solvent is evaporated from the emulsion under magnetic agitation which results in the formation of the vesicles suspended into the aqueous medium [34,36]. The solubility of the components is the main factor when choosing the phase in which they will be dissolved: lipophilic and hydrophilic compounds are added in the organic or in the aqueous phase, respectively. Besides, HLB value of non-ionic surfactants normally rules for their addition to organic or aqueous phase: the lower value, the more lipophilic, and the higher value, the more hydrophilic. Additionally, depending on the procedure, the components could be added in different phases, especially the non-ionic surfactants due to their amphiphilic character [35].

Considering all these issues, the aim of this study was to determine, on the one hand, the influence of incorporating chloroquine in niosomes as a biophysical performance enhancer agent and, on the other hand, the effect of varying non-ionic surfactant components and their phase of addition—aqueous or organic—on the biophysical performance of niosomes. For that purpose, three different niosomes, named as formulations **1**, **2** and **3**, were developed. Formulations **1** and **2** differed in one of the two non-ionic surfactants and their phase of addition and both contained chloroquine as a “helper” component. Formulation **3** was elaborated as formulation **1**, and only differed in the “helper” component as it was formulated without chloroquine. The three niosome formulations were prepared by the o/w emulsion technique and were analyzed in terms of differential scanning calorimetry and rheological properties. Niosomes were complexed with the reporter plasmid EGFP (pEGFP) to obtain nioplexes at cationic lipid/DNA mass ratios 2/1, 5/1 and 10/1, in order to characterize their mean particle size, dispersity, zeta potential and morphology. The membrane packing capacity of nioplexes was also evaluated, as well as the affinity to bind DNA, release it and protect it from enzymatic digestion. In addition, the buffering capacity of the formulations and their endosomal escape ability were also studied. Finally, in vitro biological studies were performed in order to determine the compatibility of the formulations with biological systems as well as their transfection efficiency and the duration of gene expression over time in human cystic fibrosis airway epithelial (CuFi-1) cells.

## 2. Materials and Methods

### 2.1. Preparation of Niosomes and Nioplexes

**Figure 1:**
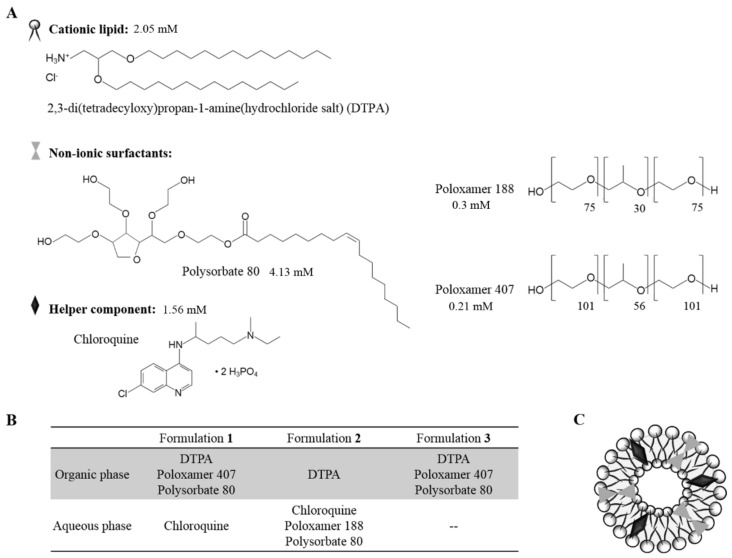
Structure and chemical components of niosomes. (**A**) Chemical structure and millimolar quantity of niosome components. (**B**) Description of the components in each formulation. (**C**) General scheme of a niosome.

Two niosome formulations based on cationic lipid were elaborated using the oil-in-water emulsion technique as previously described [18]. Both formulations **1** and **2** contained in their organic phase 5 mg of the tailor-synthesized cationic lipid 2,3-di(tetradecyloxy)propan-1-amine(hydrochloride salt) (DTPA) [37], dissolved in 1 mL of dichlorometane (DCM) (PanReac, Barcelona, Spain). The organic phase of formulation **1** also contained 12.5 mg of poloxamer 407 (Merck KGaA, Darmstadt, Germany) and 12.5 mg of polysorbate 80 (PanReac, Barcelona, Spain) as non-ionic surfactants. The aqueous phase of both formulation **1** and **2** contained 2.5 mg of chloroquine (Merck KGaA, Darmstadt, Germany) as “helper” component dissolved in 5 mL of distilled water. Furthermore, the aqueous phase of formulation **2** also contained 12.5 mg of poloxamer 188 (BASF, Ludwigshafen, Germany) and 12.5 mg of polysorbate 80 as non-ionic surfactants. Formulation **3** was prepared as to formulation **1** protocol but with the avoidance of chloroquine. The emulsion was obtained by mixing the organic and the aqueous phases by sonication (Branson Sonifier 250; Branson Ultrasonics Corporation, Danbury, CO, USA) for 30 s at 50 W. The organic solvent was removed from the emulsion by evaporation under magnetic agitation for 1 h at room temperature. The chemical structure of the components, the summary of each formulation components and the general scheme of their disposition in a niosome are represented in Figure 1. 

Nioplexes were elaborated by mixing an appropriate volume of DNA stock solution (EGFP reporter plasmid or pEGFP) with different volumes of the niosome formulations to obtain different cationic lipid/DNA mass ratios 2/1, 5/1 and 10/1. The mixture was incubated for 30 min at room temperature to enhance electrostatic interactions between the niosomes and the genetic material to form the nioplexes.

### 2.2. Plasmid Propagation

*Escherichia coli* DH5α was used to propagate the CMS-EGFP reporter plasmid (5.5 kb, PlasmidFactory, Bielefeld, Germany), named as pEGFP. Then, according to manufacturer’s instructions, the pEGFP was purified using the Qiagen endotoxin-free plasmid purification Maxi-prep kit (Qiagen, Hilden, Germany). The final concentration of pEGFP was quantified by measuring the absorbance at 260 nm using a SimpliNano™ device (GE Healthcare, Buckinghamshire, UK).

### 2.3. Nano DSC Studies

The characterization of the thermostability of niosome formulations **1**, **2** and **3** was performed by differential scanning calorimetry (DSC) using a Nano DSC device (TA Instruments, New Castle, DE, USA). Both in the reference and in the sample cells, MilliQ^®^ water was introduced in order to obtain a buffer line. Measurements of formulations **1**, **2** and **3** were performed with niosomes in the sample chamber at a concentration of 0.5 mg/mL, prior to degassing. The temperature range was from 4 °C to 100 °C for all the samples and the scan rate was 1.0 °C/1 min. Results were collected and analyzed using the DSC Run 4.6 and Launch Nano Analyze 3.11 software (TA instruments, New Castle, DE, USA), respectively. The Nano DSC chambers were cleaned after each run with MilliQ^®^ water, 2% DECON™ 90 and methanol.

### 2.4. Rheological Studies

The rheological behavior of niosomes based on formulations **1**, **2** and **3** was conducted using the Advanced Rheometer AR1000 equipment (TA instruments, New Castle, DE, USA). A flat plate with 20 mm of diameter was used. The concentration of the niosome formulations was 1 mg/mL and the GAP was settled at 1200 µm. The shear stress and the viscosity data were obtained at shear rates from 10 to 1000 s^−1^ with 10 points per decade. Data were collected and processed using the Rheology Advantage™ software (TA instruments, New Castle, DE, USA).

### 2.5. Morphology, Size, Dispersity and Superficial Charge

The morphology of niosomes was determined by transmission electron microscopy (TEM) as previously described [6]. The hydrodynamic diameter, which includes particle size, reported as mean particle intensity, and dispersity (Đ) of niosomes and their corresponding nioplexes was measured by dynamic light scattering (DLS), and the zeta potential was measured by Lasser Doppler velocimetry (LDV) in a Zetasizer Nano ZS (Malvern Instruments, Worcestershire, UK). To carry on the measurements, 50 µL of each sample were diluted in 950 µL of 0.1 mM NaCl solution. The particle hydrodynamic diameter was obtained by cumulative analysis. The Smoluchowski approximation supported the calculation of the zeta potential from the electrophoretic mobility. All measurements were carried out in triplicate.

### 2.6. Nioplexes Membrane Packing Studies

Lipid packing studies were carried out for niosomes based on formulations **1**, **2** and **3** and their corresponding nioplexes with the lipophilic probe laurdan (6-Dodecanoyl-2-Dimethylaminonaphthalene) (Fisher Scientific, Madrid, Spain). Laurdan was dissolved in dimethyl sulfoxide (DMSO) to obtain a concentration of 200 µM (stock solution). The assay was performed in a 96-well black plate. The concentration of laurdan fluorescent dye in each well was 0.5 µM and formulations at room temperature were added at a concentration of 0.25 mM. Then, 0.1 mM of NaCl was added to reach a final volume of 200 µL per well. The plate was measured in the TECAN plate reader at an excitation wavelength of 340 nm and the emission spectrum was measured from 400 to 500 nm, increasing 5 nm in each measurement and considering the intensity values at 440 nm (I_440_) and 490 nm (I_490_). The general polarization (GP) value was calculated as a relative measure for membrane order using the following formula: GP = (I_440_ − I_490_)/(I_440_ + I_490_) [38]. GP values range from −1 (least ordered) to +1 (most ordered). As a blank, laurdan reagent in NaCl solution was used. Each measurement was performed in triplicate.

### 2.7. ITC Studies

Isothermal titration calorimetry (ITC) was used to monitorize niosome-pEGFP interactions for nioplexes formation using a MicroCal PEAQ-ITC microcalorimeter (Malvern Instruments, Worcestershire, UK). The assays were carried out at 25 °C by stepwise injections of niosome formulations (1 mg/mL DTPA) into the reaction cell loaded with an aqueous solution of pEGFP (0.0166 mg/mL) at the following injection sequences: 1 × 0.4 µL; 8 × 1.7 µL; 9 × 2.5 µL (formulation **1**) and 1 × 0.4 µL; 6 × 1 µL; 14 × 2.3 µL (formulations **2** and **3**). The injections were carried out automatically under 750 rpm stirring. The heat contributed by niosome dilution in MilliQ^®^ water was measured in separate runs using the same injection sequence and subtracted from the total heat produced following each injection prior to the data analysis. The full set of experiments was carried out with the same preparation of pEGFP for the three formulations, and using the same dilution of niosomes in the binding and dilution runs of each formulation, in order to minimize errors.

### 2.8. DNA Release from Niosomes and Protection Capacity

An agarose (Merck KGaA, Darmstadt, Germany) gel electrophoresis assay was developed to analyze the ability of the niosomes to protect and release the DNA from enzymatic digestion. For DNA release assay, 12 µL of 7% sodium dodecyl sulfate (SDS) (Merck KGaA, Darmstadt, Germany) was added to the samples and incubated for 10 min at room temperature. For DNA protection analysis, 2 µL of DNase I enzyme (Merck KGaA, Darmstadt, Germany) was added to the samples and incubated for 30 min at 37 °C, then 12 µL of 7% SDS (Merck KGaA, Darmstadt, Germany) was added and incubated for 10 min at room temperature. Before running the gel, 2 µL of loading buffer were added to all samples. Naked DNA was used as a control at each condition. The amount of DNA per well was 200 ng in all cases. The agarose gel (0.8%) was immersed in a Tris-acetate-EDTA buffer and exposed for 45 min to 100 V. Once running was stopped, DNA bands were stained with GelRed™ (Biotium, Hayward, CA, USA) and images were obtained with a ChemiDoc™ MP Imaging System and analyzed with ImageLab™ Software (Bio-Rad Laboratories, Hercules, CA, USA). 

### 2.9. Buffer Capacity Assay

Acid-base titration assay was performed to determine the buffer capacity of the niosome formulations, as described previously [39]. Each sample, with 0.1 mg/mL of cationic lipid, was prepared in 10 mL of 150 mM NaCl and adjusted to pH 10 with 0.1 M NaOH. Then, the samples were titrated with 0.1 M HCl solution, added in 5 µL to 5 µL, and pH values were measured by a Crison pH-Meter GLP21.

### 2.10. Vulnerability Assay of Complexes in the Late Endosome

Micelles based on phosphatidylserine (PS) were developed as an analogue of the lysosomal compartment, as described previously [40,41]. PS was dissolved in chloroform at 1.6 mM and, straightaway, the solvent was completely evaporated under magnetic stirring. Dried sample was resuspended with phosphate buffer solution and a dispersion was obtained by sonication. PS and the nioplexes were incubated for 1 h at a pEGFP/PS mass ratio of 1/50. Naked DNA was used as control. Subsequently, the amount of the released DNA from each complex was determined by agarose gel electrophoresis. Samples (containing 200 ng of pEGFP each well) were loaded onto a 0.8% agarose gel and exposed for 30 min to 100 V. DNA bands staining was performed as aforementioned. The quantification of DNA bands was obtained using ImageLab 4.0.1 software and the percentage of DNA released was calculated applying the equation (1) where SC is supercoiled DNA:

% DNA released = (SC band/total DNA) × 100(1)

### 2.11. Cell Culture and Transfection Assays

Human cystic fibrosis airway epithelial (CuFi-1) cells obtained from ATCC^®^ CRL-4013™ were incubated at 37 °C and 5% CO_2_ atmosphere in collagen type IV (Merck KGaA, Darmstadt, Germany) pre-treated flasks/plates and were split every 2–3 days to maintain monolayer coverage. The cells were cultivated in bronchial epithelial growth medium—2 bullet kit—(BEGM) (Lonza, Basel, Switzerland). 

For transfection assays, cells were seeded in 24 well plates at a density of 1.6 × 10^5^ cells per well (for posterior flow cytometry analysis) or in 96 well plates at a density of 3 × 10^4^ cells per well (for posterior kinetic analysis) and incubated overnight to achieve 70% of confluence at the time of transfection. The formation of nioplexes at 2/1, 5/1 and 10/1 cationic lipid/DNA mass ratios were performed in serum-free Opti-MEM transfection medium (Gibco, San Diego, CA, USA). The growth medium was removed from the plate and the cells were exposed to nioplexes (1.25 µg and 0.21 µg of pEGFP per well in 24-well and 96-well plates, respectively) for 4 h in the incubator. After the incubation, nioplexes were removed and fresh growth medium was added to the cells. As a negative control for transfection, cells were not exposed to nioplexes but were incubated in Opti-MEM for 4 h. As a positive control for transfection, Lipofectamine™ 2000 transfection reagent (Invitrogen, Waltham, MA, USA) was used. Each condition was performed in triplicate. 

### 2.12. Analysis of EGFP Expression and Cell Viability

Transfection capacity and compatibility with biological systems of nioplexes **1**, **2** and **3** were evaluated over 7 days both qualitatively and quantitatively, using the Cytation™ 1 equipment (BioTek Instruments, Winooski, VT, USA). For quantitative analysis of transfection efficiency and biocompatibility, the green fluorescence intensity and the cell absorbance at 600 nm were measured every 24 h for 7 days. For qualitative analysis of both parameters, brightfield and fluorescent images of cells were also acquired every 24 h for 7 days. Cells were kept alive for the whole experiment and incubated at 37 °C and 5% CO_2_ in the Cytation™ 1 equipment, without retrieving or moving the plate between measurements. 

Transfection efficiency and cell viability were further quantitatively evaluated 48 h after the exposure to nioplexes by flow cytometry. Specifically, EGFP expression, cell viability and mean fluorescence intensity (MFI) analysis were conducted using a FACSCalibur system flow cytometer (Becton, Dickinson and Company, Franklin Lakes, NJ, USA). Transfected cells were washed with Dulbecco´s phosphate buffered saline (DPBS) with calcium and magnesium (Lonza, Basel, Switzerland). Cells were detached using Trypsin/EDTA 0.25% (Gibco, San Diego, CA, USA) and, then, trypsin inhibitor (DPBS with 1% of fetal bovine serum (Gibco, San Diego, CA, USA) was added. Cells were centrifuged at 1100 rpm for 5 min and the resulting pellet was resuspended in culture medium and transferred to specific flow cytometer tubes. In order to evaluate cell viability, propidium iodide (Merck KGaA, Darmstadt, Germany) was added in each sample at 1:300 dilution. The fluorescent signals were measured at 525 nm (FL1) and 650 nm (FL3) corresponding to EGFP positive cells and dead cells, respectively. To establish a collection gate and exclude cells debris, non-transfected cells, used as control samples, were displayed on a forward scatter (FSC) vs. side scatter (SSC) dot plot. Positive transfection control samples containing Lipofectamine™ 2000 transfected cells were used to establish cytometer settings and channel compensations. Cell viability data were normalized in relation to the value of non-transfected control cells. For each sample, 10,000 events were collected. The experiments were carried out in triplicate for each condition. 

### 2.13. Statistical Analysis

The statistical analysis was carried out using the IBM SPSS Statistics 25 software. The Shapiro–Wilk test was used to evaluate normal distribution, and the Levene test was used to evaluate homogeneity of variance. In parametric conditions, Student´s *t* test or ANOVA followed by the post-hoc HSD Tukey test was performed. In non-parametric conditions, the Kruskal–Wallis test and/or the Mann–Whitney *U* test for unpaired comparisons was performed. In all cases, *p* value ≤ 0.05 was considered statistically significant. Data were represented as mean ± standard deviation (SD).

## 3. Results

### 3.1. Characterization of the Thermostability of Niosomes

**Figure 2:**
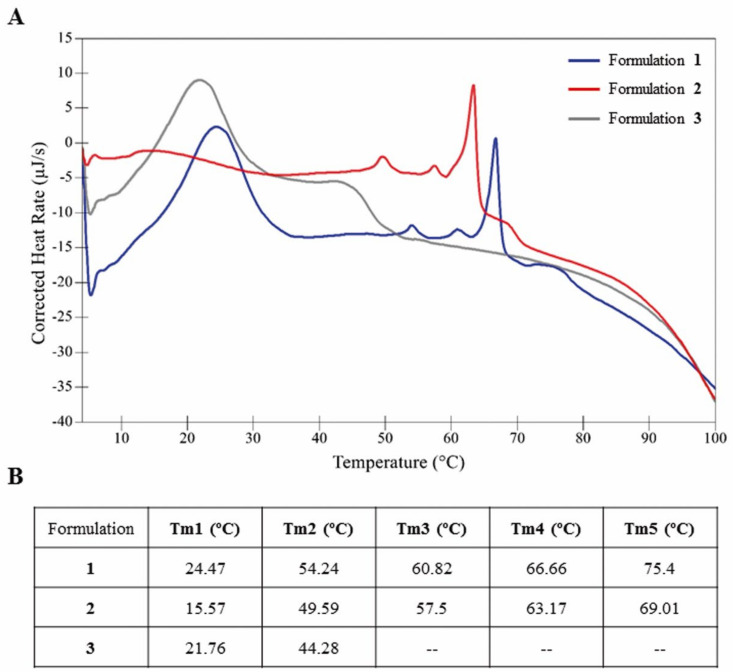
(**A**) Thermograms of niosomes based on formulations **1** (blue line), **2** (red line) and **3** (grey line) in aqueous solution. Scan rate: 1.0 °C/1 min. (**B**) Thermal melting temperature (Tm) data for the transitions of niosome formulations.

The thermostability of niosome formulations **1**, **2** and **3** was evaluated by differential scanning calorimetry. As shown in Figure 2, the thermogram of formulation **1** (blue line) was slightly shifted to the right compared to formulations **2** (red line) and **3** (grey line), indicating higher thermal stability than its counterparts (Figure 2A). Regarding their thermal melting temperatures (Tm), formulation **1** showed five well-defined peaks, while in the case of formulation **2** a first wide and weak peak around 15 °C followed by four well-defined peaks were obtained. In the case of formulation **3** only two clear peaks were reported (Figure 2B). Remarkably, although slightly shifted to the right, formulation **1** coincided with formulation **3** in Tm1 and with formulation **2** in Tm2, Tm3, Tm4 and Tm5 (Figure 2A,B).

### 3.2. Rheological Properties of Niosomes

**Figure 3:**
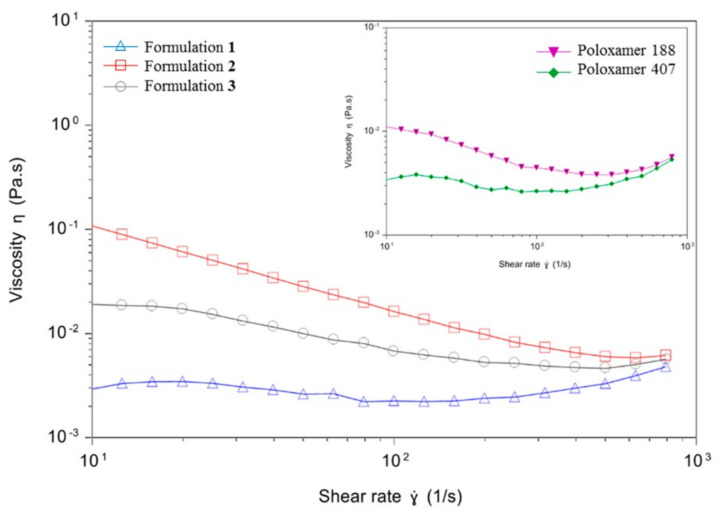
Rheology measurements. Main graph: viscosity curves of niosomes based on formulations **1** (blue line, triangles), **2** (red line, squares) and **3** (grey line, dots) expressed as a function of shear rate. Secondary graph: viscosity curves of poloxamer 188 (violet line, inverted triangles) and poloxamer 407 (green line, rhombus) expressed as a function of shear rate.

Rheological studies were performed in order to analyze the viscosity of niosomes based on formulations **1**, **2** and **3** as a function of the shear rate (Figure 3). Formulation **1** (blue line, triangles) showed the lowest viscosity values among the three formulations, and they remained quite stable when increasing the shear rate, indicating a Newtonian rheological behavior. On the contrary, formulations **2** (red line, squares) and **3** (grey line, dots) showed the higher initial viscosity values which declined when incrementing the shear rate, denoting a pseudoplastic rheological behavior. Regarding the rheological properties of the non-ionic surfactants employed in niosome formulations, poloxamer 188 (violet line), used to elaborate formulation **2**, showed higher viscosity values than poloxamer 407 (green line), used to elaborate formulations **1** and **3**.

### 3.3. Morphology, Size, Dispersity and Superficial Charge

**Figure 4:**
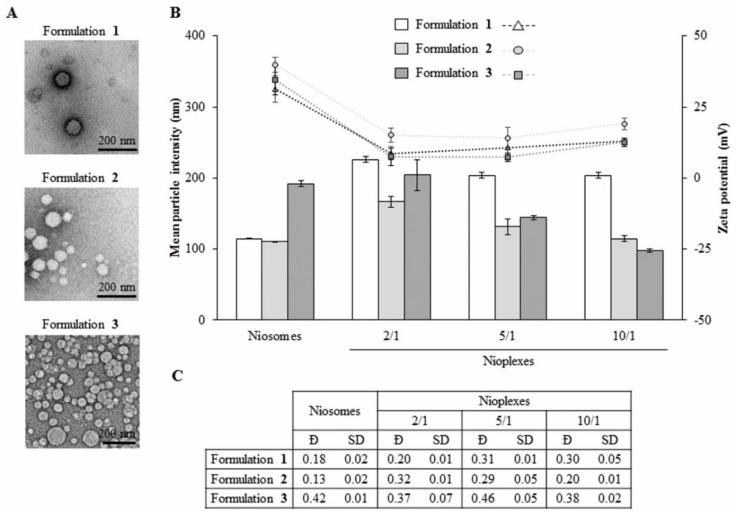
Characterization of niosomes based on formulations **1**, **2** and **3** and their corresponding nioplexes vectoring EGFP plasmid (pEGFP) at cationic lipid/DNA mass ratios 2/1, 5/1 and 10/1. (**A**) Transmission electron microscopy (TEM) images. Scale bar: 200 nm. (**B**) Mean particle intensity (bars) and zeta potential (symbols) values of niosomes and their corresponding nioplexes represented by the mean ± SD of three measurements. (**C**) Dispersity (Đ) values of niosomes and their corresponding nioplexes. Each value represents the mean ± standard deviation (SD) of three measurements.

Niosome formulations **1**, **2** and **3** showed a clear spherical shape without particle aggregation (Figure 4A). The mean particle size of niosome formulations **1**, **2** and **3** were 114.43 ± 0.64 nm, 110.40 ± 0.40 nm and 191.73 ± 4.11 nm, respectively (Figure 4B, bars). When complexing to pEGFP at the cationic lipid/DNA mass ratio 2/1, nioplexes based on formulation **1** showed a 2-fold increase in mean particle size, while nioplexes based on formulation **2** presented a more restrained increase of around 1.5-fold and nioplexes based on formulation **3** did not present a significant increase. When increasing the cationic lipid/DNA mass ratios to 5/1 and 10/1, mean particle sizes declined progressively, with a more pronounced slope in the case of nioplexes based on formulations **2** and **3**. In all cases, the mean size values of nioplexes based on formulation **1** were higher than the values based on formulations **2** and **3**. Zeta potential data of niosome formulations **1, 2** and **3** were +31.37 ± 4.78 mV, +39.93 ± 2.64 mV and +34.63 ± 5.28 mV, respectively (Figure 4B, dots). After the addition of DNA, an initial decrease was observed at the cationic lipid/DNA mass ratio 2/1 and then values increased again with increasing cationic lipid/DNA ratios without reaching the original zeta potential of niosomes in all cases. Regarding dispersity (Ð), formulations **1** and **2** showed low values below 0.35, while formulation **3** had higher values both with and without DNA (Figure 4C).

### 3.4. Nioplexes Membrane Packing Studies

**Figure 5:**
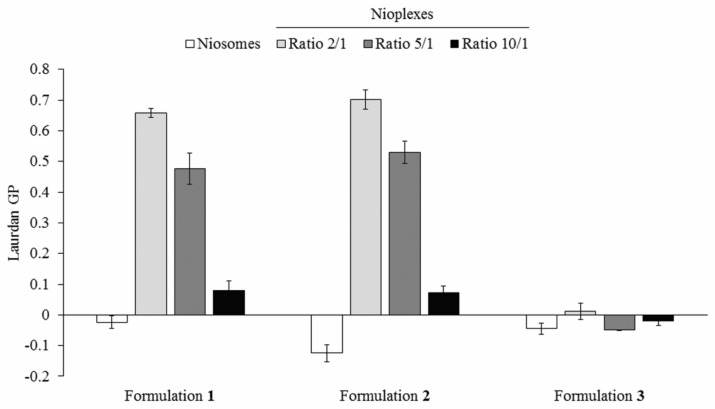
Laurdan general polarization (GP) values measured of niosomes based on formulations **1**, **2** and **3** and their corresponding nioplexes at cationic lipid/DNA mass ratios 2/1, 5/1 and 10/1.

Membrane GP values were determined in niosomes based on formulations **1**, **2** and **3** their corresponding nioplexes. As shown in Figure 5, formulation **1** niosomes showed negative GP value close to zero that increased at 2/1 and 5/1 cationic lipid/DNA mass ratio to values near 0.7 and 0.5, respectively. Such values decreased again to values around zero at 10/1 mass ratio values. Formulation **2** followed a similar pattern, with the lowest negative value in niosome formulation and, with higher, positive values at ratios 2/1 and 5/1 and a pronounced decrease to values near zero at the cationic lipid/DNA mass ratio 10/1. Formulation **3** presented an intermediate negative value in the niosome formulation and the lowest GP values near zero that switched to negative values at cationic lipid/DNA mass ratios 2/1, 5/1 and 10/1.

### 3.5. Evaluation of Niosome-pEGFP Interactions by ITC

**Figure 6:**
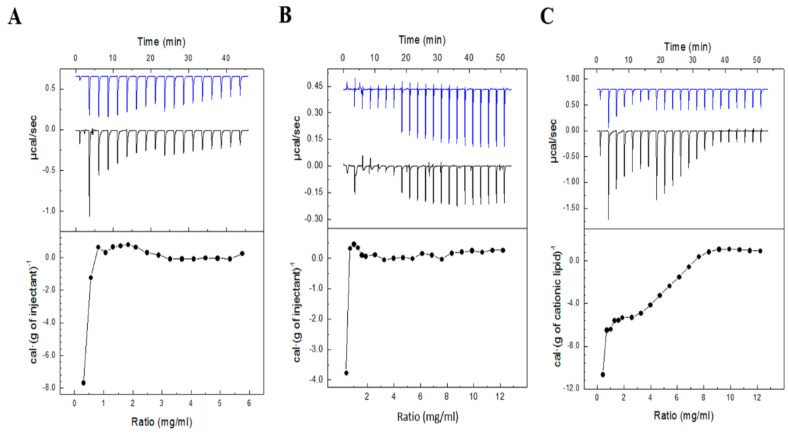
Isothermal titration calorimetry (ITC) of (**A**) formulation **1** (1:1 dilution), (**B**) formulation **2** and (**C**) formulation **3** into pEGFP. Upper panels show the raw data for the injection of respective formula into the plasmid solution (black line) or the blank solution (blue line). Lower panels show the dependence of the heat evolved by gram of cationic lipid injected as a function of the DTPA/pEGFP mass ratio in the sample cell (measures were carried out at 25 °C).

The interactions between DNA and niosome formulations **1**, **2** and **3** were followed by ITC, and the heat evolved per gram of DTPA injected as a function of the cationic lipid/DNA mass ratio is shown in Figure 6. Results showed that the titration profiles of niosomes **1** and **2** followed a similar trend, while a clear change was observed in the titration profile of formulation **3**, lacking chloroquine. 

### 3.6. Buffer Capacity and Endosomal Escape of Nioplexes

**Figure 7:**
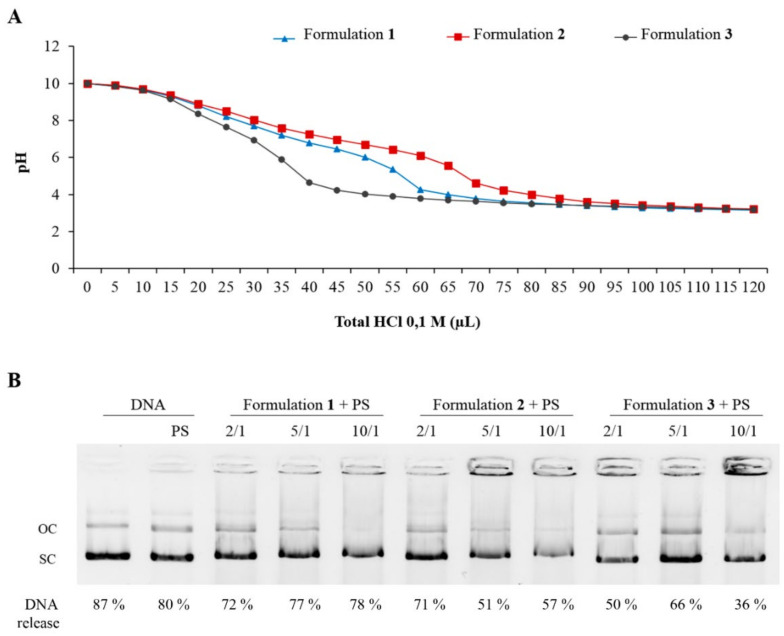
Buffer capacity and endosomal escape evaluation. (**A**) Analysis of pH buffering capacity of niosomes based on formulations **1** (blue line, triangles), **2** (red line, squares) and **3** (grey line, dots). (**B**) DNA release profiles in agarose gel electrophoresis assay of nioplexes based on niosome formulations **1**, **2** and **3** at cationic lipid/DNA mass ratios at 2/1, 5/1 and 10/1 from phosphatidylserine (PS) micelles. OC: open circular. SC: supercoiled.

In order to determine the ability to escape from intracellular endosomes, the buffer capacity and the DNA release profile from artificial endosomes of nioplexes based on formulations **1**, **2** and **3** were evaluated. Among the three niosomes, no significant differences were observed between formulations **1** (blue line, triangles) and **2** (red line, squares), while formulation **3** (grey line, dots) showed the lowest buffering capacity (Figure 7A). Regarding the ability of formulations to escape from endosomal compartment analogues based on PS, results showed that formulations released the DNA after the contact with the lipid membrane of the PS micelles, especially formulation **1**, which revealed the highest ability to escape from artificial endosomes at all cationic lipid/DNA mass ratios evaluated (Figure 7B). 

### 3.7. Cell Viability and Transfection Efficiency of Nioplexes

**Figure 8:**
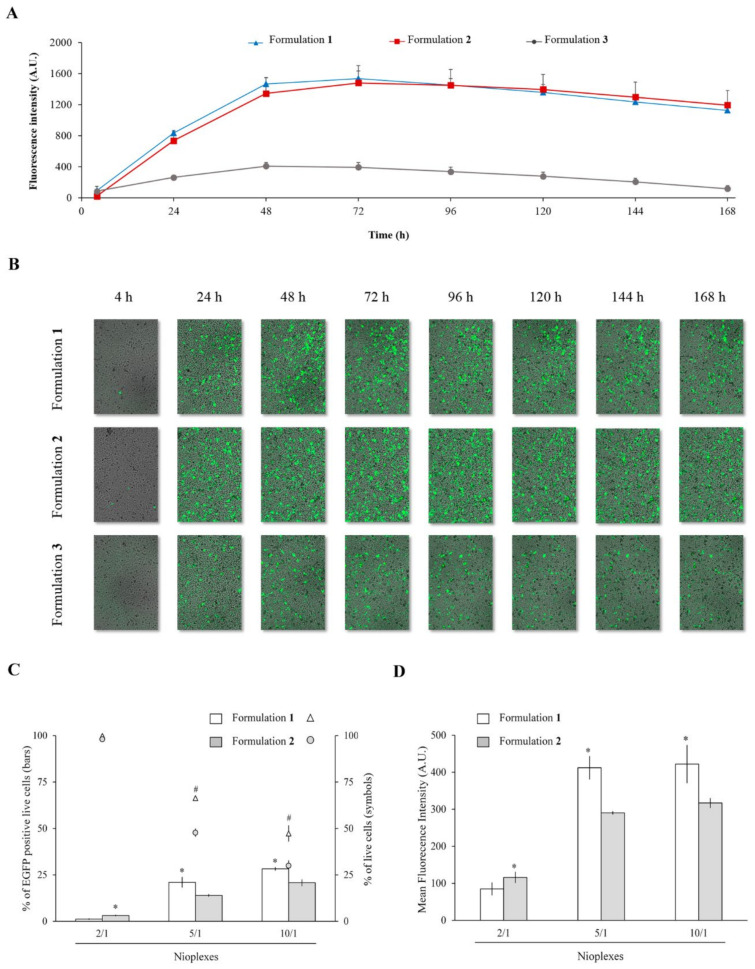
Transfection capacity and cell viability assays. (**A**) Fluorescence intensity over time in CuFi-1 cells transfected with nioplexes based on formulation **1**, **2** and **3** at cationic lipid/DNA mass ratio 5/1. Each value represents the mean ± SD of three measurements. (**B**) Fluorescence microscope images showing EGFP positive CuFi-1 cells transfected with nioplexes based on formulation **1**, **2** and **3** at cationic lipid/DNA mass ratio 5/1 and cellular appearance over time. (**C**) Percentages of EGFP positive live cells (bars) and cell viability (symbols) measured by flow cytometry in CuFi-1 cells 48 h after transfection with nioplexes **1** and **2** at cationic lipid/DNA mass ratios 2/1, 5/1 and 10/1. Each value represents the mean ± SD of three measurements. (**D**) Mean fluorescence intensity values. Each value represents the mean ± SD of three measurements. Statistical significance: * *p* ≤ 0.05 in transfection and MFI; # *p* ≤ 0.05 in cell viability.

Transfection and cell viability assays were performed during 7 days in CuFi-1 cells with nioplexes based on formulations **1**, **2** and **3** vectoring pEGFP at cationic lipid/DNA mass ratios 2/1, 5/1 and 10/1. Although the highest ratio 10/1 sometimes achieved higher transfection efficiency, the best results in terms of desirable balance between efficiency and cell viability were obtained with the intermediate mass ratio 5/1 (Figure S1). Focusing on the cationic lipid/DNA mass ratio 5/1, formulations **1** and **2** showed similar fluorescence intensity values, and both reached the maximum fluorescence peak 72 h after transfection (Figure 8A, blue and red lines, respectively). After 72 h, the fluorescence intensity values declined slightly, but were relatively stable until the 7th day. Formulation **3** revealed lower fluorescence intensity values than its counterparts all over the 7 days of the experiment, and reached its maximum value 48 h after transfection (Figure 8A, grey line). Figure 8B shows representative images of CuFi-1 cells transfected with nioplexes **1**, **2** and **3** at the cationic lipid/DNA mass ratio 5/1 over time, with no or little transfection at 4 h and evident increase of EGFP positive cells from 24 h after transfection. Differences on the amount of EGFP positive cells between cells exposed to formulations **1** or **2** and to formulation **3** are also visible in these images. Finally, in order to quantify more precisely the differences in transfection efficiency and cell viability between nioplexes based on formulations **1** and **2**, further flow cytometry assays were conducted. Results showed that, at cationic lipid/DNA mass ratios 5/1 and 10/1, the percentage of EGFP expressing cells was significantly (*p* ≤ 0.05) higher with nioplexes based on formulation **1** (21.02 ± 2.68% and 28.22 ± 0.76%, respectively) than with those based on formulation **2** (13.94 ± 0.56% and 20.75 ± 1.68%, respectively) (Figure 8C, bars). Regarding cell viability, formulation **1** also showed significantly (*p* ≤ 0.05) higher percentages of live cells than formulation **2**, although in both cases values decreased when increasing the cationic lipid/DNA mass ratios (Figure 8C, dots). These results were further confirmed by the MFI values, where at cationic lipid/DNA mass ratios 5/1 and 10/1, formulation **1** also showed higher values than formulation **2** with significant differences (values around 400 vs. values around 300, respectively) (Figure 8D). Lipofectamine™ 2000 was used as a transfection positive control and showed 34.07 ± 2.52% of EGFP expressing live cells, 65.36 ± 1.29% of cell viability and a MFI value of 626.34 ± 26.30 (data not shown).

## 4. Discussion

In this work we determined, on the one hand, the influence of incorporating chloroquine and, on the other hand, the effect of varying non-ionic surfactant components and their phase of addition—aqueous or organic—on the biophysical performance of niosome formulations **1**, **2** and **3**. As it is well known by scientific community, chloroquine is a chemical compound that can promote the endosomal escape and enhance the transfection efficiency [42]. However, as far as we are concerned, how chloroquine affects the biophysical performance and correlate with their transfection efficiency of niosome formulations has not been assessed until now. It has also been well described that variations on chemical structure and composition, biophysical properties and preparation methods of lipid-based non-viral vectors can affect to their gene delivery efficiency and cytotoxicity both in vitro and in vivo [43]. In this respect, some works have described how liposomal formulations with different manufacturing process carrying amphotericin B resulted in significant differences in terms of physicochemical properties, efficiency and toxicity, despite the fact of presenting the same components in their formulations [44,45,46]. Additionally, the manufacturing processes required for the scale-up production of such formulations in order to reach clinical practice could also affect to their biological performance. To avoid this scenario, the European Medicines Agency (EMA) and the Food and Drug Administration (FDA) specified the requirements required to obtain the bioequivalence of liposomal formulations [47] and compiled precise guidance documents for the complete characterization of nanosystems in order to guarantee the safety and efficacy of the new drugs based on nanomaterials [48,49]. All these requirements could also be applied to niosome formulations in order to track the manufacturing process as well as to evaluate their properties and activity, considering that they could represent an attractive alternative to liposomes with better properties for gene therapy purposes. 

Regarding the chemical structure and composition of the niosome formulations **1**, **2** and **3** developed in the present work, the compounds used have been previously reported for nucleic acid delivery. In particular, the cationic lipid DTPA, which contains two saturated hydrocarbonated chains, a glycerol backbone and an amino as cationic head [18], has shown suitable properties for gene therapy [18,41,50,51]. As non-ionic surfactants polysorbate 80 and poloxamer 188 and 407 were included. Polysorbate 80 is one of the most employed non-ionic surfactant in niosomes because avoids nanoparticle aggregation, decreases the toxicity frequently associated to cationic lipids and improves the transfection efficiency due to its polyethylene glycol (PEG) chains [7,50,52]. Poloxamer 188 can prevent and repair membrane disruption and is useful to stabilize the lipid bilayer of niosomes [53,54,55], thereby improving the gene transfer efficiency [56]. Poloxamer 407 has been used in micro- and nanoparticles to stabilize and prolong the half-life of formulations as well as to prevent from aggregation [57,58] and to enhance the transfection process [59]. Regarding the HLB values of these non-ionic surfactants, poloxamer 188 (HLB = 28) has the highest value, followed by poloxamer 407 (HLB = 18–22) and, lastly, by polysorbate 80 (HLB = 15). Nevertheless, because of their amphiphilic nature, all of them could be incorporated either in the aqueous or in the organic phase during the preparation of nanoemulsions [39]. As “helper” component, formulations **1** and **2** included chloroquine due to its capacity to protect and interact with the DNA [60,61]. Moreover, chloroquine improves the endosomal escape impairing the fusion of endosomes and lysosomes because of its protonation inside the vesicles, causing a higher pH value that avoids the enzymatic lysosomal activity [30,62,63,64,65]. Therefore, in the present work, we hypothesized that chloroquine would not only act as a “helper” component, but also as a biophysical performance enhancer agent inside the niosomes. For this purpose, an exhaustive biophysical characterization is described in order to stablish its potential correlation with their biological activity.

First, niosome formulations **1**, **2** and **3** were prepared by the o/w emulsion technique and analyzed by DSC in order to determine their thermal stability and associated structural transitions. The Tm analysis, which corresponds to the maximum peak of endothermal events [66], showed relevant differences among the three formulations. Such differences are commonly found in lipid nanoparticle dispersions with various components affecting molecular packing, which is reflected in the different melting points and enthalpies [67]. The “fine structure” of the thermogram in the region between 48 °C and 70 °C of formulations **1** and **2** is clearly associated with the effect caused by the chloroquine component on the disposition of lipids within the niosome. This effect could be related, on the one hand, to the higher packing shown by the lipids in both formulations and, on the other hand, to their higher transfection efficiency. The removal of chloroquine from formulation **3** caused disappearance of the well-defined transitions observed in that region and, instead, a possible transition between 40 °C and 50 °C was observed. On the other hand, the shift towards higher temperatures observed in formulation **1** could be due not only to the chloroquine content (formulation **1** vs. formulation **3**), but also to the substitution of poloxamer (188 in formulation **2**, by 407 in formulation **1**), and the incorporation of such surfactants in the organic phase (formulation **1**) instead of in the aqueous phase (formulation **2**). This could be related to its ability to stabilize the formulations, meaning that higher temperatures are needed in order to induce structural alterations in formulation **1** compared to its counterparts as indicated by the noticeable shift to the right of its thermogram [68]. In addition, the transition centered at 21.76 °C (formulation **3**) or 24.47 °C (formulation **1**) is possibly associated with the presence of poloxamer 407, since in formulation **2** a wide and low intensity transition is observed instead, which was centered around 15–16 °C and which could be due to the presence of poloxamer 188. 

Next, rheological studies were conducted in order to evaluate the flow behavior of the niosome formulations. Formulation **2** appeared to be the most viscous among the three formulations, with values that clearly declined when increasing the shear rate, as it is common in solutions with a pseudoplastic rheological behavior [69]. Formulation **3** showed a similar pseudoplastic behavior, with lower viscosity values. Interestingly, formulation **1** showed the lowest and more constant viscosity values, which would indicate a Newtonian rheological behavior [69]. In this regard, the major viscosity values of formulation **2** could be in part attributed to its non-ionic surfactant poloxamer 188, which has higher viscosity values than poloxamer 407 used in the preparation of formulations **1** and **3**. Therefore, these results suggested that the substitution of poloxamer 407 by poloxamer 188 and, probably, also its incorporation in the aqueous phase instead of the organic phase, increased the viscosity and affected to the rheological behavior of the formulation. On the other hand, the differences found between formulations **1** and **3** could only be due to the presence of chloroquine, as it is the only difference between both formulations. In this sense, the incorporation of chloroquine in formulation **1** significantly affects the arrangement of the lipid membrane, as evidenced by DSC studies and GP values (see below), modifying its rheological behavior. Taken together, rheological analysis suggests that the low viscosity value of formulation **1** could contribute, among many other physicochemical factors, to the highest transfection efficiency reported by this formulation. Such viscosity values would maintain constant at different shear stress due to the Newtonian rheological behavior [70].

Subsequently, in order to further understand the differences between the three formulations and their applicability for gene delivery purposes, niosomes **1**, **2** and **3** were complexed to the reporter pEGFP at cationic lipid/DNA mass ratios 2/1, 5/1 and 10/1 to obtain their corresponding nioplexes. Their morphology, size, dispersity and zeta potential were studied, parameters that provide insights about the transfection capacity of the formulations. The size of all niosomes and nioplexes ranged between 100 and 230 nm, which it is appropriate to enhance the cellular uptake of the nanoparticles [71]. The positive zeta potential values of the three niosomes enhance electrostatic interactions with the plasmid DNA and, therefore, the nioplexes formation [72]. Additionally, these high positive values prevent particle aggregation and improve cellular internalization [73]. As predicted, surface charge data decreased after the addition of the genetic material due to the partial neutralization of positive charges of the cationic lipid amine groups by the negatively charged phosphate groups of the DNA [74]. Regarding dispersity, formulations **1** and **2** revealed narrow size distributions, as indicated by their low values, while formulation **3** showed higher values, indicating a more heterogeneous particle size distribution than its counterparts [7], which could be due to the lack of chloroquine. 

The biophysical properties studied are closely related to another relevant parameter, specifically, to the niosome membrane packing. Therefore, we studied the membrane environment in niosome formulations **1**, **2** and **3** and their corresponding nioplexes vectoring pEGFP at cationic lipid/DNA mass ratios of 2/1, 5/1 and 10/1, by measuring the GP values. In niosomes measurements, the highest value was obtained by formulation **1** and the lowest by formulation **2**. The nioplexes values showed clear differences of formulations **1** and **2** respect to formulation **3**, especially at cationic lipid/DNA mass ratios of 2/1 and 5/1. Results revealed a more disordered membrane lipid packing profile in formulation **3** compared to formulations **1** and **2** after complexing with pEGFP, which showed GP values similar to the lipid packing values found in model membrane [75]. The highest membrane lipid packing profile was obtained by nioplexes based on formulations **1** and **2** with chloroquine content, yielding more condensed complexes than formulation **3**, devoid of chloroquine. In addition, the increase obtained in GP values from niosomes to nioplexes based in formulation **1** and **2**, could indicate that some ordered packing was formed via interaction of pDNA with the niosomes components, suggesting a relevant role of chloroquine in the packing of nioplexes possibly due to its ability to interact with the DNA [60,61]. Moreover, slight variations between formulations **1** and **2** could be attributed to the different poloxamer non-ionic surfactants content, since poloxamer 407—used to prepare formulation **1**–, presents longer alkyl chains than poloxamer 188—used to prepare formulation **2**–, which is often related to higher rigidity and, therefore, could contribute to increase the membrane packing of nioplexes based on formulation **1**. As indicated above, membrane packing is also related with other biophysical parameters studied in this work, such as thermal stability, viscosity and the rheological behavior of the formulations which, all together, could affect to the gene delivery process. In fact, it has been described that the presence of the drug astaxanthin in liposomes affects to the thermodynamic, viscoelastic and electrical properties of lipid membranes [76]. In our case, it could be assumed that the incorporation of chloroquine in the niosome formulation increases the membrane lipid packing of nioplexes as well as the thermal stability of formulations and, ultimately, enhances the transfection efficiency of these niosome formulations.

The effect of chloroquine incorporation in the interaction of niosome formulations with DNA was further supported by ITC results. Titration of niosome formulations into DNA showed that formulations **1** and **2** reached to a plateau or saturation point at DTPA/pEGFP mass ratios around 2/1‒3/1, whereas saturation with formulation **3** required a mass ratio of 9/1, which suggests a less binding affinity for DNA molecules. Therefore, it can be concluded that the addition of chloroquine increased the DNA binding affinity of niosome formulations, which is in accordance with previous reports [4] and would also in part explain the higher transfection efficiency of these formulations compared to formulation **3**. Additionally, ITC results suggested that variations reported in the structural and functional properties of nioplexes based in formulations **1** and **2** at mass ratios above 2/1 likely reflect a redistribution of DNA leading to nioplexes with a decreasing fraction of plasmid molecules bound as niosome concentration was further increased. Besides, the titration results were in accordance with the results obtained from nioplexes packing since the presence of chloroquine promoted greater order of niosome membrane packing. Once characterized the capacity to bind DNA, agarose gel electrophoresis assays were performed to evaluate the ability to protect and release the genetic material from enzymatic degradation (Figure S2). Results showed that the three formulations were able to protect the genetic material against enzymatic degradation at cationic lipid/DNA mass ratios 5/1 and 10/1, but not at the lower mass ratio 2/1, suggesting that this ratio would not be suitable for gene delivery purposes. For subsequent cell transfection assays, we selected the cationic lipid/DNA mass ratio 5/1 in all formulations, because it reported enough capacity to protect the genetic material and contributed more than the higher ratio 10/1 to mitigate the cytotoxic effect sometimes associated to high amounts of cationic lipids [77]. 

The last parameters that were evaluated before moving on to biological assays were the buffer capacity and the endosomal escape ability from artificial PS micelles (mimicking cellular endosomes) of the niosome formulations. Considering that DNA quantity loaded in all wells was the same, the differences observed in the percentages of released DNA from nioplexes could be explained by the different chemical composition of the formulations. Among the three niosomes, formulation **3** showed the lowest buffering capacity and the lowest ability to escape from artificial endosomes. This suggests that the incorporation of chloroquine in formulations **1** and **2** might enhance the endosomal escape via the proton sponge effect, which is a widely used strategy in formulations with a high buffering capacity, as is the case of these two formulations [14,30]. However, other possible endosomal escape mechanisms such as pore formation in the endosomal membrane, flip-flop mechanisms or fusion in the endosomal membrane mechanisms, among others, could also contribute to the endosomal escape behavior observed [78,79,80]. The ability to release the DNA once the formulations contact the lipid membrane of the endosomal compartment is essential for an efficient transfection process [41]. 

Finally, the biological performance of nioplexes **1**, **2** and **3** at cationic lipid/DNA mass ratio 5/1 was studied in CuFi-1 cells, because of the autosomal monogenic recessive condition of cystic fibrosis disease, which makes it particularly attractive for future gene therapy applications using niosomes as non-viral vectors [81]. In particular, the cell viability after exposure to nioplexes, the transfection efficiency of the formulations and the duration of gene expression were evaluated. Results clearly showed that the presence of chloroquine was necessary to achieve high transfection levels and, in addition, formulations with this component were well tolerated by the cells as indicated by the healthy cellular appearance along 7 days in cells exposed to formulations **1** and **2**. Generally, chloroquine is considered to be cytotoxic at concentrations superior to 100 µM, while it has been reported to enhance endosomal escape of polymeric nanoparticles at a concentration of 75 µM [82]. However, in this work cells were exposed to a concentration of chloroquine of 24.23 µM, which was enough to enhance endosomal escape and far away from the cytotoxic concentration. Considering that most niosome formulations enter the cells via endocytic pathways, which usually end in late endosomes, the ability to promote endosomal escape is essential in order to reach high transfection efficiencies [7]. Therefore, the endosomal escape observed in formulations **1** and **2** would in part explain the higher transfection efficiency of chloroquine-containing niosomes compared to formulation **3**, devoid of chloroquine.

Regarding the duration of gene expression, the fluorescence intensity values reached their maximum 48–72 h after transfection and then were maintained quite stable or declined slightly over time. Interestingly, the gene expression was maintained over seven days, which affects to the design of specific dosage regimens for gene therapy applications. Hence, the kinetics evaluation of transgene expression employing formulations **1**, **2** and **3**, corroborated the key role of chloroquine in this process. In order to further analyze the influence of non-ionic surfactants and the inversion of the addition phase on the biological performance of formulations **1** and **2**, additional more accurate flow cytometry studies were carried out at 48 h post-transfection [7]. Results showed significant better cell tolerance as well as higher EGFP positive cell percentages and MFI values in cells treated with formulation **1**, revealing this formulation as the best of the three evaluated for gene delivery purposes. This could be attributed to the specific biophysical advantages shown by formulation **1**, including its superior thermal stability and a Newtonian rheological behavior, as well as to other non-considered parameters such as the cell entry pathways or the cellular uptake capacity. Therefore, it could be deduced that not only the chloroquine content, but also the non-ionic surfactant chemical composition and protocol of incorporation also affects to the transfection efficiency of niosome formulations, although further research is needed in order to elucidate the exact mechanism of such effect. Hence, formulation **1**, which contains chloroquine in the aqueous phase and surfactants incorporated in the organic phase, might be an encouraging non-viral strategy for gene therapy aimed at cystic fibrosis disease. In this regard, previous in vivo studies carried out by our group showed a successful gene delivery capacity of formulation **1** in central nervous system [62], but an in depth biophysical study and implementation in congenital disease model, as cystic fibrosis, was a missing issue.

## 5. Conclusions

In conclusion, the main findings of the present study are obtained from the comparative evaluation, on the one hand, of formulations **1** and **2**, which differed on the composition of non-ionic surfactant and on the phase of addition of those components and, on the other hand, of formulations **1** and **3**, which only differed in the presence or absence of the helper component chloroquine. Differences observed on formulations **1** and **3** clearly revealed the importance of chloroquine content in niosome vesicles since affected not only to physicochemical parameters but also to the biological performance of niosomes. Overall, results revealed that chloroquine improved thermal stability, lowered viscosity and reduced particle size and dispersity values. In addition, chloroquine content improved DNA binding affinity and membrane packing organization in corresponding nioplexes. Such relevant parameters along with the enhanced buffering capacity could explain, at least in part, the highest transfection efficiency values reported when chloroquine was incorporated into the niosome formulations. The increased capacity of such niosomes that contain chloroquine to escape from artificial endosomes could also contribute to improve gene delivery efficiency. Thus, chloroquine emerges as an interesting material able to improve the biophysical properties and the transfection efficiency of niosomes for non-viral gene therapy applications. On the other hand, the effect of both chemical composition and protocol of incorporation of non-ionic surfactants (formulation **1** vs. formulation **2**), resulted in subtle biophysical variations, although formulation **1** showed better thermal stability, lower viscosity values with Newtonian rheological behavior and higher transfection efficiency. Taken together, these results support the requirements of the regulatory agencies for the complete characterization of nanoparticles aimed for biomedical applications and scaling-up. Of note, the biophysical parameters evaluated for full physicochemical and biological characterization of niosomes and their corresponding nioplexes, could be used as gold standard for further gene therapy nanosystems evaluation. Hence, the present work provides an in depth analysis of different biophysical parameters and characterization strategies that are relevant for nanosystems in gene delivery purposes and that might be interesting to include in the specifications of regulatory agencies for the evaluation of new drugs based on nanomaterials.

## Supplementary Materials

The following are available online at https://www.mdpi.com/article/10.3390/pharmaceutics13111787/s1, Figure S1: Transfection efficiency and cell viability assays in CuFi-1 cells transfected with nioplexes based on formulation **1** (F1), **2** (F2) and **3** (F3) at cationic lipid/DNA mass ratios 2/1, 5/1 and 10/1. Figure S2: Protection and SDS-induced release of DNA in niosome formulations **1**, **2** and **3** complexed to pEGFP at cationic lipid/DNA mass ratios 2/1, 5/1 and 10/1 visualized by agarose gel electrophoresis.
