# Supplementary material for: Correlation between Biophysical Properties of Niosomes Elaborated with Chloroquine and Different Tensioactives and Their Transfection Efficiency
Source: Pharmaceutics. 2021 Oct 26;13(11):1787. doi: 10.3390/pharmaceutics13111787 (PMC8623750; doi:10.3390/pharmaceutics13111787)
Supplement: Supplementary file 1 [file pharmaceutics-13-01787-s001.zip › pharmaceutics-1424135-supplementary.pdf]

pharmaceutics

MDPI

Supplementary Material: Correlation between biophysical properties of niosomes elaborated with chloroquine and different tensioactives and their transfection efficiency

Myriam Sainz-Ramos, Ilia Villate-Beitia, Idoia Gallego, Nuseibah AL Qtaish, Margarita Menéndez, Laura Lagartera, Santiago Grijalvo, Ramón Eritja, Gustavo Puras, José Luis Pedraz

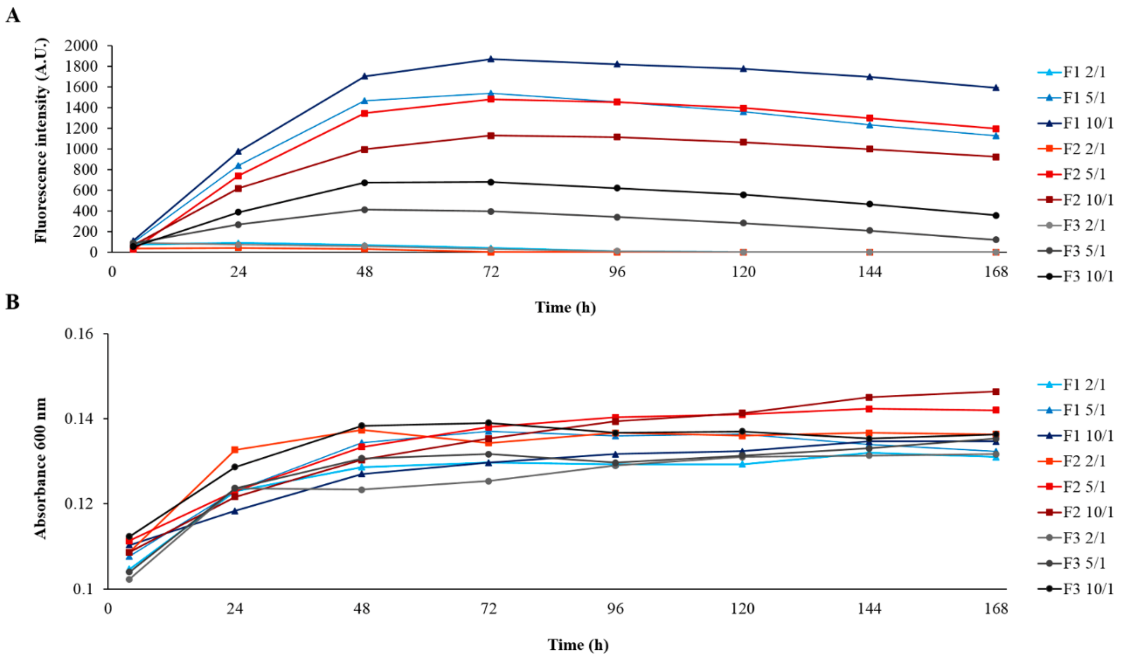

Figure S1. Transfection efficiency and cell viability assays in CuFi-1 cells transfected with nioplexes based on formulation 1 (F1), 2 (F2) and 3 (F3) at cationic lipid/DNA mass ratios 2/1, 5/1 and 10/1. (**A**) Fluorescence intensity over time. Each value represents the mean ± SD of three measurements. (**B**) Mean absorbance curves over time. Each value represents the mean ± SD of three measurements.

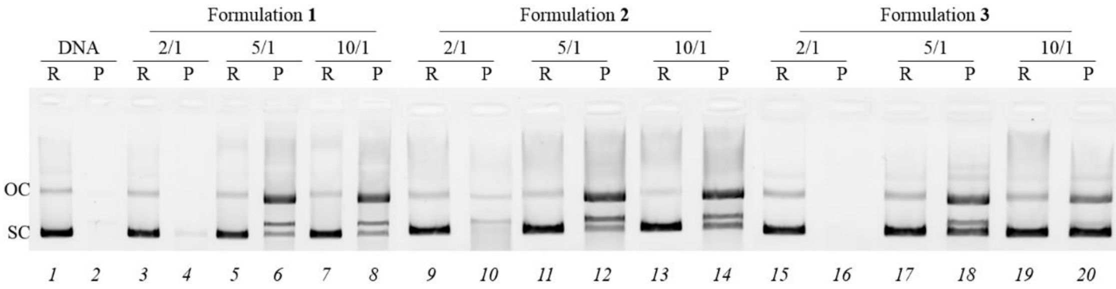

Figure S2. Protection and SDS-induced release of DNA in niosome formulations **1**, **2** and **3** complexed to pEGFP at cationic lipid/DNA mass ratios 2/1, 5/1 and 10/1 visualized by agarose gel electrophoresis. Lanes 1–2 correspond to naked DNA; lanes 3–4 to formulation **1** at ratio 2/1; lanes 5-6 to formulation **1** at ratio 5/1; lanes 7-8 to formulation **1** at ratio 10/1; lanes 9-10 to formulation **2** at ratio 2/1; lanes 11–12 to formulation **2** at ratio 5/1; lanes 13–14 to formulation **2** at ratio 10/1; lanes 15–16 to formulation **3** at ratio 2/1; lanes 17–18 to formulation **3** at ratio 5/1; lanes 19–20 to formulation **3** at ratio 10/1. Nioplexes were treated with SDS (lanes 3, 5, 7, 9, 11, 13, 15, 17 and 19) and DNase I + SDS (lanes 4, 6, 8, 10, 12, 14, 16, 18 and 20). Upon the addition of SDS, naked DNA (lane 1) completely migrated in the gel, and so did the DNA complexed to the formulations at cationic lipid/DNA mass ratios 2/1, 5/1 and 10/1 as indicated by the intense supercoiled (SC) signal (lanes 3, 5, 7, 9, 11, 13, 15, 17 and 19). After the addition of the enzyme DNase I, no DNA signal was observed in the case of naked DNA (lane 2) and very faint or no signal was neither observed in the case of DNA complexed to formulations **1**, **2** and **3** at cationic lipid/DNA mass ratio 2/1 (lanes 4, 10 and 16, respectively). However, the intense DNA bands observed with DNA complexed to formulations **1**, **2** and **3** at cationic lipid/DNA mass ratio 5/1 (lanes 6, 12 and 18, respectively) as well as with DNA complexed to formulations **1**, **2** and **3** at cationic lipid/DNA mass ratio 10/1 (lanes 8, 14 and 20, respectively), indicated that the genetic material was protected and released by the formulations at those higher cationic lipid/DNA mass ratios. R: DNA release. P: DNA protection. OC: open circular. SC: supercoiled.
